# Hepatitis C virus and Human Immunodeficiency Virus coinfection among attendants of Voluntary Counseling and Testing Centre and HIV follow up clinics in Mekelle Hospital

**DOI:** 10.11604/pamj.2013.14.107.2302

**Published:** 2013-03-18

**Authors:** Haftom Hadush, Solomon Gebre-Selassie, Adane Mihret

**Affiliations:** 1 Department of Microbiology, Immunology and Parasitology, School of Medicine, Wollega University, P.O.Box 395, Nekemte, Ethiopia; 2 Department of Microbiology, Immunology and Parasitology, School of Medicine, Addis Ababa University, Ethiopia; 3 Armauer Hansen Research Institute, P.O.Box 1005, Addis Ababa, Ethiopia

**Keywords:** Hepatitis C virus, human immunodeficiency virus, co-infection, seroprevalence

## Abstract

**Introduction:**

Hepatitis C virus remains a large health care burden to the world. HIV and HCV coinfection is major global health concern worldwide. However, there is limited information on the prevalence of HCV/HIV co-infection in Ethiopia. The aim of the study was to assess the magnitude of HIV/HCV coinfection and the potential risk factors in attendants of voluntary counseling and testing centre and HIV follow up clinics of Mekelle hospital.

**Methods:**

A cross sectional seroprevalence survey of HCV infection was carried out on 300 HIV negative and positive subjects attending voluntary counseling and testing (VCT) center and HIV follow up clinics of Mekelle hospital, Ethiopia from December 2010-February 2011. Serum samples were tested for anti-HCV antibodies using immunochromatographic test.

**Results:**

Of the 300 study participants, 126(42%) were HIV negative and 174(58%) HIV seropositive from VCT and HIV follow up clinics, respectively. The overall anti-HCV prevalence was 18(6.0%). There were no significant differences in HCV seroprevalence among the different categories of age and sex (p> 0.05). Of the 174 persons with HIV, 16 (9.2%) cases had antibodies to HCV, where as among 126 HIV negative subjects 2 (1.58%) were HCV seropositive (p= 0.006, OR= 6.28, 95% CI= 1.42-27.82).

**Conclusion:**

Accordingly, there was a significant difference in sero-positivity of HCV between HIV positive and HIV negative participants. No apparent risk factor that caused HCV infection was inferred from this study.

## Introduction

Hepatitis C virus (HCV) is a significant healthcare problem affecting more than 170 million people worldwide and as many as four million new infections occur annually [1] HCV may increase the rate of progression to acquired immune-deficiency syndrome (AIDS), impair immune reconstitution and the risk of hepa-toxicity. HCV infection increases the number of complications in persons who are co-infected with human immune-deficiency virus (HIV) [2]. Of those exposed to HCV, 80% become chronically infected, and at least 30% of the carriers develop chronic liver disease [3]. Risk factors associated with HCV infection include injection drug use, receipt of blood products, long term haemo-dialysis, organ transplantation, receipt of tattoo from unsanitary facilities, vertical transmission during pregnancy and sexual exposure [4]. The WHO estimated that about 3% of the world′s population is chronically infected with HCV [5]. The overall prevalence in Sub-Saharan Africa is estimated 3% [6]. Decline of mortality due to opportunistic infections in HIV-infected patients since the introduction of highly active antiretroviral therapy (HAART) has led to an increase in morbidity and mortality related to hepatitis virus infections. Co-infection of HIV and HCV is common because of their similar routes of transmissions of exposure. The sero-prevalence of HCV in Ethiopia was reported as 7.5% [7] and HIV/HCV co-infection rate was 1.4%-11.6% [8–10]. Screening of blood products is the only way to prevent transfusion associated cases. Diagnostic tests for HCV infection include serologic tests to detect HCV antibodies, molecular tests and genotyping techniques [11]. Screening based on antibody detection has markedly reduced the risk of transfusion-related infection [12]. The aim of the study was to assess the magnitude of HIV/HCV co-infection rate and potential risk factors are associated with HCV sero-positivity among consecutive attendants of voluntary counseling and testing (VCT) centre and HIV follow up clinics of Mekelle hospital.

## Methods

Study population, data collection and laboratory procedures.

The prospective study was conducted during December 2010 to February 2011among attendants of VCT centre and ART clinic in Mekelle hospital, North Ethiopia. Three hundred subjects (126 HIV-negative from VCT and 174 HIV-positive subjects in ART follow up) were enrolled in the study. Socio-demographic data including risk behaviors, drug injection, dental procedure, catheterization, surgery, blood transfusion, hospitalization, history of tattooing, scarification and bloodletting were collected. Five millilitre of blood sample was collected by finger prick from consecutive attendants of VCT and ART clinics in the hospital. Serum was screened for anti-HCV antibody using rapid test kits (Flavicheck-HCV WB, Qualpro diagnostics, India) and one step HCV serum test strip (Biocare TM diagnostics, China). The reactive sera were further tested by Enzyme-Linked Immuno-sorbent assay (ELISA) for confirmation. HIV and anti-HCV positive samples were retested by 4^th^ generation ELISA assay (HCV antibody ELISA, Human diagnostics, Germany). The presence of antibodies to HIV was determined using three different immune-chromatography rapid test kits: HIV (1+2) Rapid Test Strip (Shanghai Kehua Bioengineering co., Ltd), HIV ½STAT-PAK Assay (Chembio Diagnostic Systems, inc.) and Uni-Gold HIV (Trinity biotech plc, Ireland). Drops of blood were taken by finger-prick using pipette. About 40% of the sample was added to the HIV (1+2) rapid test cassette and then one drop of the sample diluents was added to the same area. After 2-3 min, one band if negative or two bands if positive were observed. When the anti-HIV was positive by the HIV Rapid Test from, the sample was retested by the second HIV ½ STAT-PAK assay. HIV-seropositive subjects were counseled and negative sera were retested by the second Rapid Test and confirmed by Uni-Gold HIV test. Rapid test reactive specimens were retested using 4^th^ generation ELISA assay.

### Data analysis

Data were analyzed using SPSS 17.0 statistical software. Chi-square (X^2^) test was utilized to compare variables. P-value <0.05 was considered as significant. Odds ratio (OR) and 95% confidence interval (CI) were used to measure the strength of association.

### Ethical considerations

Ethical clearance was obtained from Department of Microbiology, School of Medicine of Addis Ababa University. Informed written consent was obtained from study participants.

## Results

Of the 300 participants, 181(60.3%) were females (male to female ratio was 0.6:1). The mean age of the participants was 28.95±9.4 (range: 6 months to 63 years). The majority, (53.0%) were adults aged 20-29 year of age. Children 60 years age were 1.3% (Table 1).

**Table 1:** HCV prevalence by age and sex in attendants of VCT centre and HIV follow up clinics, Mekelle hospital

Age group (years)	HCV-Antibody positive No (%)		Total No (%)
	Males	Females	
0-9	0(0.0)	0(0.0)	0(0.0)
10-19	0(0.0)	1(25.0)	1(9.1)
20-29	2(3.9)	7(6.5)	9(5.7)
30-39	2(6.5)	3(5.5)	5(5.8)
40-49	0(0.0)	2(25.0)	2(8.7)
50-59	1(14.3)	0(0.0)	1(12.5)
>60	0(0.0)	-	0(0.0)
**Total**	**5(4.2)**	**13(7.2)**	**18(6.0)**

Of the 300 study participants tested for anti-HCV, 135(74 females, 61 males) were from VCT centre and 165(107 females, 58 males) were HIV positive cases from ART clinic in Mekelle hospital. Among the 174 HIV positive subjects, 16(9.2%) were positive for HCV antibody. The prevalence of HCV infection among HIV negative VCT centre attendants was 2/126(1.6%). The overall prevalence of anti-HCV antibody was 18(6.0%) {95%CI, 3.6-9.3}. The age specific prevalence was higher 1/8(12.5%) for the 50-59 year age groups. Males positive for anti-HCV were 5(4.2%) while females were 13(7.2%). HCV infection was higher in females than males (OR, 0.57; 95%CI, 0.20-1.63, P=0.33) (Table 1).

The overall prevalence of HIV/HCV co-infection in HIV positive patients was 16(9.2%). Of the 174 persons with HIV, 5/62(8.1%) were males and 11/112(9.8%) females. Among 126 HIV-negative subjects, 2(1.6%) females were positive for HCV-antibody. The proportion of HCV infection in HIV cases was increased nearly 6 fold compared to HIV negative individuals which was 9.2% and 1.6%, respectively (OR, 6.28; 95%CI, 1.42-27.82, P=0.006) (Table 2). The age specific pattern of HIV and HCV co-infection shows that the frequency of HCV infection was in similar trend to the frequency of HIV infection in each age group of the study subjects.

**Table 2:** Coinfection of HIV and HCVin attendants of VCT centre and HIV follow up clinics, Mekelle hospital

HIV status	HCV antibody		Total No (%)	OR, 95%CI	P value
	Positive No (%)	Negative No (%)			
Positive	16(9.2)	158(90.8)	174	6.28(1.42-27.82)	0.006
Negative	2(1.6)	124(98.4)	126		
**Total**	18(6.0)	282(94.0)	300(100)		

A relatively higher proportion of HCV infection was observed in respondents with history of multiple sex partners and using tattoo, 6/59(10.2%) and 3/35(8.6%), respectively. A total of 2(8.3%) respondents had history of admission to hospital, 1/17(5.9%) had history of dental procedure (Table 3).

**Table 3:** HCV prevalence by risk factors in attendants of VCT centre and HIV follow up clinics, Mekelle hospital

Risk factors	HCV-antibody, No (%)			OR (95% CI)	p value
	Positive	Negative	Total		
**Community acquired**					
Tattooing	3(8.6)	32(91.4)	35(14.0)	1.56(0.43-5.69)	0.45
Blood letting	0(0.0)	8(100.0)	8(3.2)	-	1.00
Scarification	1(3.1)	31(96.9)	32(12.8)	0.48(0.06-3.70)	0.70
**Hospital acquired**					
Hospitalization	2(8.3)	22(91.7)	24(9.6)	1.48(0.32-6.84)	0.65
Blood transfusion	0(0.0)	6(100.0)	6(2.4)	-	1.00
Dental procedure	1(5.9)	16(94.1)	17(6.8)	0.98(1.22-7.82)	1.00
Minor surgery	0(0.0)	16(100.0)	16(6.4)	-	0.61
**Behavioral acquired**					
Multiple sex partners	6(10.2)	53(89.8)	59(23.6)	2.16(0.78-6.02)	0.14
STI/STD	0(0.0)	30(100.0)	30(12.0)	-	0.23
Abortion	0(0.0)	23(100.0)	23(9.2)	-	0.38
Total	13(5.2)	237(94.8)	250(100.0)		

Regarding the various occupational groups, 2(11.1%) of 18 farmers had positive HCV-antibody and 5/50(10.0%) of office workers had HCV prevalence, while daily laborers had 4/43(9.3%) prevalence (Table 4).

**Table 4:** HCV prevalence by occupation in attendants of VCT centre and HIV follow up clinics, Mekelle hospital

Occupational category	HCV- antibody (%)		Total
	Positive	Negative	
Health workers	0(0.0)	2(100.0)	2(0.7)
Sex workers	1(6.7)	14(93.3)	15(5.3)
Day laborers	4(9.3)	39(90.7)	43(15.4)
Office workers	5(10.0)	45(90.0)	50(17.9)
Handicrafts	0(0.0)	12(100.0)	12(4.3)
Farmers	2(11.1)	16(88.9)	18(6.4)
Merchants	0(0.0)	1(100.0)	1(0.4)
Housewives	3(3.6)	80(96.4)	83(29.6)
Drivers	0(0.0)	7(100.0)	7(2.5)
No job	2(4.1)	47(95.9)	49(17.5)
**Total**	**17(6.1)**	**263(93.9)**	**280(100.0)**

## Discussion

Sero-prevalence of HCV among HIV positive individuals of 16(9.2%) is higher than HIV-negative of 2(1.6%). This result is higher than the 0.7% report in HIV-positive subjects by Tessema et al [13] and 1.7% by Gelaw and Mengistu [14]. Lower HIV/HCV co-infected rates were reported from Gambia [15], Nigeria [16], South Africa [17] and Uganda [18], in 0.6%, 1.86%, 1.9% and 3.3%, respectively. In India, prevalence of HCV-antibody in HIV-positive patients showed 1.6% [19]. In Nigeria, another study showed 2.3% HCV- antibody co-infection in HIV-positive patients [20] while in Greece 7.5% [21]. In a similar study carried out in Iran, the co-infection rate of HCV in HIV-positive patients was 74% [22]. Findings of 8.2% in Nigeria [23] and 8.6% in Cameroon [24] were reported which were comparable with the present study. In contrast, the co-infection rate obtained in the present study 9.2% is lower than 11.6% reported by an earlier study in VCT and HIV follow up clinic [8]. This was lower than co-infection rate of 30-50% reported some industrialized countries, such as in North America and Europe [25, 26], where intravenous drug use (IDU) is a major risk factor for both infections. The high co-infection rate in HIV-positive persons could demonstrate that patients may be exposed to HCV infection by sexual contact and it suggests co-transmission of both viruses [9].The frequency of HCV transmission to sexual partners is significantly higher when HIV virus is also transmitted. This would suggest that HIV could be co-factor for the sexual transmission of HCV infection [27]. Therefore, investigation of HCV in HIV-positive patients is vital in order to take care of them [28]. This finding agrees with other studies in where HCV infection was significantly higher in HIV infected cases than in HIV negative individuals 11.6% vs 2.6% [8]. In addition, Tessema *et al* [13] reported the presence of a significant association between HCV and HIV infections. The possible elucidation for this variation might be due to the rapid test positive samples in the present study was not confirmed by other tests.

The overall prevalence of HCV infection among HIV negative persons was 2/126(165%). This result is lower than the 5% rate observed among VCT attendants reported by Gebre [8]. Injection drug use is uncommon in the study area. Regarding occupational groups, 2/8(11.1%) of farmers were positive for HCV-antibody and office workers had HCV prevalence of 5/50(10.0%), while day laborers had 4/43(9.3%) prevalence. In another study, HCV-antibody distribution with respect to occupation was reported uniform [8]. Seroprevalence rates of HCV antibody was higher in individuals with multiple sex partners and in sex workers suggesting that sexual transmission may be possible.

This study has several limitations. Supplemental anti-HCV testing (i.e., RIBA) for all anti-HCV positive confirms the presence of anti-HCV. The presence of screening test negative result is common in immune-suppressed individuals (HIV infection) and during window period of the disease. Hence, these assays do not exclude the possibility of exposure to HCV virus for the individuals with negative results.

## Conclusion

Co-infection rate of HIV/HCV in this study is high. Thus, diagnosing HCV in HIV-positive patients is vital in order to take care of them and allot resources in health planning. Preventive measures against HCV should target primarily on HIV infected people. Therefore, providing opportunity of screening for HCV infection in all HIV-infected patients is essential. Implementation of more effective public health education and counseling are essential to reduce the dangers of HIV/HCV co-infection.
